# Quest for the Molecular Basis of Improved Selective Toxicity of *All-Trans* Isomers of Aromatic Heptaene Macrolide Antifungal Antibiotics

**DOI:** 10.3390/ijms221810108

**Published:** 2021-09-18

**Authors:** Julia Borzyszkowska-Bukowska, Justyna Górska, Paweł Szczeblewski, Tomasz Laskowski, Iwona Gabriel, Jakub Jurasz, Katarzyna Kozłowska-Tylingo, Piotr Szweda, Sławomir Milewski

**Affiliations:** Department of Pharmaceutical Technology and Biochemistry and BioTechMed Centre, Gdańsk University of Technology, 80-233 Gdańsk, Poland; julborzy@student.pg.edu.pl (J.B.-B.); justyna.m.gorska@gmail.com (J.G.); pawel.szczeblewski@pg.edu.pl (P.S.); tomasz.laskowski@pg.edu.pl (T.L.); iwogabri@pg.edu.pl (I.G.); jakjuras@student.pg.edu.pl (J.J.); katarzyna.kozlowska-tylingo@pg.edu.pl (K.K.-T.); piotr.szweda@pg.edu.pl (P.S.)

**Keywords:** aromatic polyene antifungals, selective toxicity, molecular modelling

## Abstract

Three aromatic heptaene macrolide antifungal antibiotics, Candicidin D, Partricin A (Gedamycin) and Partricin B (Vacidin) were subjected to controlled *cis-trans* *→ all trans* photochemical isomerization. The obtained *all-trans* isomers demonstrated substantially improved in vitro selective toxicity in the *Candida albicans* cells: human erythrocytes model. This effect was mainly due to the diminished hemotoxicity. The molecular modeling studies on interactions between original antibiotics and their photoisomers with ergosterol and cholesterol revealed some difference in free energy profiles of formation of binary antibiotic/sterol complexes in respective membrane environments. Moreover, different geometries of heptaene: sterol complexes and variations in polyene macrolide molecule alignment in cholesterol-and ergosterol-containing membranes were found. None of these effects are of the crucial importance for the observed improvement of selective toxicity of aromatic heptaene antifungals but each seems to provide a partial contribution.

## 1. Introduction

Polyene macrolide antibiotics produced by *Streptomyces* spp. demonstrate antifungal activity. They are characterized by a large macrocyclic lactone ring (macronolide) containing a system of 3–7 conjugated double bonds and a number of hydrophilic substituents, including a glycosidically bound aminosugar, most often D-mycosamine (3,6-dideoxy-3-amino-d-mannose) [1]. The most interesting and prospective group of polyene macrolides are the heptaenes, with Amphotericin B (AmB) still being considered as ‘the lifesaving drug’ and ‘the golden standard’ for antifungal agents [2]. Heptaenes fall into two major categories: non-aromatic heptaenes (including AmB) and aromatic heptaene macrolides (AHMs). The latter contain the *p*-aminoacetophenone moiety attached to an aliphatic sidechain and exhibit the *cis-trans* geometry of the heptaene chromophore, in contrast to *all-trans* configuration in non-aromatic heptaenes [3].

The molecular mechanism of antifungal action of polyene macrolide antibiotics has been most studied for Amphotericin B, although it is generally accepted that for all these compounds, their antifungal activity is due to the specific interactions with sterols present in the cell membranes. According to the most widely accepted “barrel-stave-pore” hypothesis on the mode of action of AmB, 4 to 12 AmB:ergosterol complexes self-assemble to transmembrane channel-like forms. The hydrophilic inner core of the AmB/ergosterol transmembrane channel provides an aqueous pore which causes leakage of cytosol components and ultimate cell death [4]. Recently, Dong et al. provided experimental evidence for vertical orientation of AmB molecules in the cell membrane [5], thus challenging an alternative “sterol sponge” model of AmB action, in which antibiotic molecules are laterally adsorbed onto membrane surface and extract ergosterol from the membrane [6], recently claimed to be valid for all glycosylated polyene macrolides, including AHMs [7]. AmB and other polyene macrolides form also complexes with cholesterol present in mammalian membranes and this ability is the reason for their mammalian toxicity. Due to the different geometries of ergosterol and cholesterol molecules, complexes with cholesterol are formed at slightly higher AmB concentrations [8]. Nevertheless, a selective toxicity of AmB, under in vitro conditions quantitatively characterized by the EH_50_/MIC ratio (where EH_50_, erythrocyte hemolysis, is the concentration causing 50% hemolysis of human erythrocytes and MIC, minimal inhibitory concentration, is the minimal drug concentration inhibiting fungal growth), is poor. It was found that AmB at concentrations about an order of magnitude higher than MIC, self-associate to form dimers or higher oligomers, that are equally toxic to mammalian and fungal cells, whereas AmB monomers, dominating at concentrations close to MIC, are selectively fungicidal [9]. Several attempts have been made to improve selective toxicity of AmB by its chemical modifications, mainly at the amino group of mycosamine or the carboxyl functionality, resulting in disturbance of self-association ability [10,11,12,13,14].

AHMs, like Vacidin (Partricin B), Gedamycin (Partricin A) or Trichomycin B, exhibit similar to AmB antifungal activity but their selective toxicity is even poorer, due to the high hemotoxicity [15]. We assumed that the hemotoxicity of AHMs, possibly resulting from their high affinity to cholesterol, might be due to the *cis-trans* geometry of the heptaene system. We also hypothesized that conversion of original *cis-trans* AHMs into their AmB-like *all-trans* isomers may afford compounds demonstrating better selective toxicity. In this paper, we show that the *all-trans* isomers of Candicidin D, Partricin A and Partricin B, indeed demonstrate an improved selective toxicity. These compounds have been obtained upon photochemical isomerization of original antibiotics. This conversion has been performed under conditions previously established for the methyl ester of 3′-*N*-acetylcandicidin complex [16]. Some elements of molecular basis for this phenomenon have been identified in consequence of molecular modelling studies. 

## 2. Results

### 2.1. Preparation and Confirmation of Identity of Candicidin D, Partricin A, Partricin B and Their All-Trans Isomers 

Three antifungal antibiotics, namely Candicidin D (CndD), Partricin A (ParA, syn. Gedamycin) and Partricin B (ParB, syn. Vacidin) have been selected as representatives of AHMs. Amphotericin B (AmB) was used as a reference compound.

**Figure 1:**
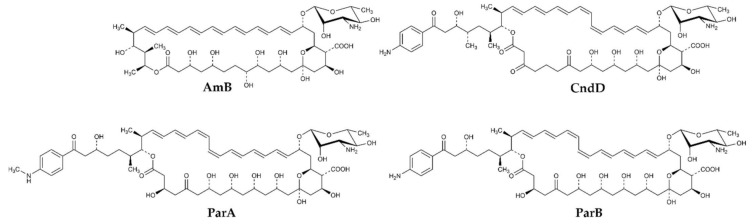
Structures of heptaene macrolide antifungal antibiotics studied in this work; AmB (Amphotericin B), CndD (Candicidin D), ParA (Partricin A), ParB (Partricin B).

CndD is the main component of Candicidin complex produced by *Streptomyces griseus* IMRU 3570, while ParA and ParB, are produced by *Streptomyces aureofaciens*, as the Aureofacin complex. All these three AHMs exhibit the *cis-trans* geometry of the heptaene fragment, with two double bonds of Z geometry located at positions 26 and 28 in CndD and at positions 28 and 30 in ParA and ParB (Figure 1). Production of these antibiotics as components of complexes (mixtures) of closely related compounds calls for selective, high-resolution separation methods. In this case, centrifugal partition chromatography (CPC) appeared highly effective for separation of ParA and ParB but in isolation of CndD from the multicomponent Candicidin complex, CPC was followed by preparative HPLC. Finally, all three compounds were obtained with purity exceeding 95%.

*All-trans* isomers of CndD, ParA and ParB, called here as *iso*CndD, *iso*ParA and *is*oParB, respectively, were obtained upon irradiation of Candicidin and Aureofacin complexes by monochromatic light of wavelength λ = 365 nm. The complexes were dissolved in a mixture of 95% methanol 5% water at 5 mg mL^−1^ and exposed to light for 2.5–3 h. Components of the post-reaction mixtures were separated by means of CPC and preparative HPLC. *Iso*CndD, *iso*ParA and *iso*ParB were finally isolated with purity exceeding 92%, while 2–6% of CndD, ParA and ParB, not converted into *all-trans* isomers, were present in the final preparations.

HPLC chromatograms of the final preparations CndD, ParA, ParB, *iso*CndD, *iso*ParA and *iso*ParB are presented as Figure S1A–F.

Identity of CndD, ParA, ParB, *iso*CndD, *iso*ParA and *iso*ParB was confirmed by ^1^H NMR, 2D ^1^H DQF-COSY (Double Quantum Filtered-Correlation Spectroscopy) NMR, mass spectrometry (MS) and UV-Vis spectroscopy. UV-Vis spectra and MS data are presented in HPLC chromatograms (Figure S1A–F), complete ^1^H NMR spectra are presented as Figure S2A–F and the most informative fragments of DQF-COSY spectra are presented as Figure S3A–F. All these data, particularly molecular peaks in MS spectrum with m/z values equal to M + 1 of each compound, differences in relative intensities of the longest wavelength absorption peaks, their batochromic shift ~6 nm in UV-Vis spectra, and characteristic patterns of signals in DQF-COSY spectra, confirmed that *iso*CndD, *isoParA* and *iso*ParB are *all-trans* isomers of CndD, ParA and ParB, respectively.

### 2.2. Antifungal In Vitro Activity, Hemolytic Properties and Selective Toxicity of CndD, ParA, ParB, isoCndD, isoParA and isoParB 

Antifungal in vitro activity of three *cis-trans* AHMs and their *all-trans* isomers was determined in two growth media, minimal RPMI-1640 and complex BDS, using the serial dilution microplate method and compared to that of AmB as a reference compound. Determination in RPMI-1640 medium was made under conditions recommended by CLSI (Clinical & Laboratory Standards Institute) [17]. The BDS medium was included for comparative purposes, since it was used in previous studies on biological properties of AHMs [15]. Eight species of the Candida genus and non-pathogenic *S. cerevisiae* yeasts were used as the test microorganisms. The results of these determinations are presented in Table 1 and Table 2.

The MIC values determined in RPMI-1640 medium were for all seven polyene macrolides markedly higher than those determined in BDS (0.06–1.0 µg mL^−1^ range vs. 0.001–0.25 µg mL^−1^ range).

The MIC values of CndD, ParA and ParB determined in BDS were generally lower than those of AmB, consistently with the previously obtained data [15]. Comparing MICs obtained for CndD and *iso*CndD, they were in most cases the same or slightly lower for the *all-trans* isomer, although *C. famata* was somewhat less susceptible to *iso*CndD. On the other hand, the *all-trans* isomers of ParA and ParB were in most cases less active than their *cis-trans* counterparts, nevertheless still more active than AmB.

In RPMI-1640 medium, *iso*CndD was equally or slightly more active than CndD against all test yeasts, except for *C. albicans*. *Iso*ParA and *iso*ParB were mostly equally or slightly less active than their ParA and ParB counterparts. Nevertheless, the differences in MIC values between respective *cis-trans* and *all-trans* isomers were not high (4 times maximally) but all six AHMs were in most cases, but not all, superior to AmB.

Data presented in Table 3 clearly show, that hemolytic activity of *all-trans* isomers of CndD, ParA and ParB was lower than that of the mother, *cis-trans* antibiotics. The degree of enhancement of the EH_50_ values was the lowest for the CndD/*iso*CndD pair (a little more than twice) but for the ParA/*iso*ParA and ParB/*iso*ParB pairs it was remarkable, more than 10-fold. All the *all-trans* isomers were much less hemolytic than AmB.

Based on the results of antifungal and hemolytic activity determination, one may calculate the selective toxicity indexes (STIs), as the EH_50_ to MIC ratio. Results of these calculations, in which we have applied the MIC values determined against *C. albicans* in RPMI 1640 medium, are presented in Table 4.

The STI values of *all-trans* isomers were always better, e.i., higher than those of the mother *cis-trans* compounds. For the CndD/*iso*CndD pair, the difference was relatively small but in the case of the ParA/*iso*ParA and ParB/*iso*ParB pairs it was much higher, thus reflecting the similar trends in hemolytic activity of these compounds. Amphotericin B appeared more selective than ParA and ParB but less selective than CndD. All the *all-trans* isomers were more selective than AmB.

It is worth mentioning that differences in STI values would be much more spectacular, if the MIC values determined against *C. albicans* in BDS medium were taken into account. Particularly, for CndD, *iso*CndD and AmB, the STIs would be 2510, 5557 and 57, respectively. Nevertheless, we believe that the antifungal activity in RPMI-1640 medium, composition of which mimics that of low molecular weight compounds fraction of human plasma, better reflects the physiological conditions of disseminated fungal infection.

### 2.3. Molecular Modeling of AH:Sterol Interactions

Basing on the stereochemistry of Gedamycin [18] and Vacidin [19,20], supplemented by the previously unknown absolute configuration at C41 [publication pending], and considering complete structural data on Candicidin D [21] and *iso*Candicidin D [16], molecular models of native (*cis-trans*) ParA, ParB and CndD compounds were prepared, as well as of their isomeric (*all-trans*) derivatives. Those molecules were individually inserted into properly prepared lipid bilayer environments, where their interactions with cholesterol (Chol) and ergosterol (Erg) were examined using computational techniques. The obtained results were referred to the data acquired for AmB, for which similar computational experiments were performed simultaneously. It must be noted that analogous molecular modelling studies on AmB were conducted before [8], yet using different phospholipid as a major membrane component (DPPC vs. DMPC). Therefore, for the sake of methodological coherence, it was decided to perform those calculations as well.

**Figure 2:**
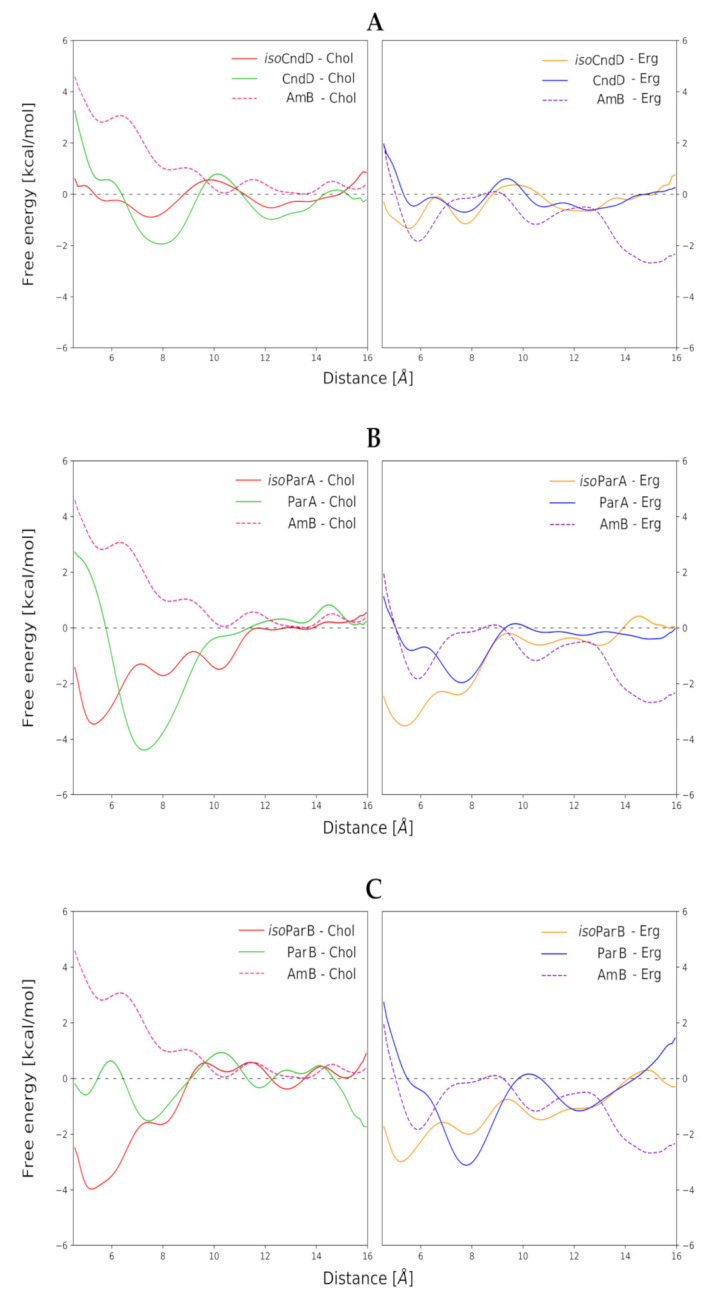
Free energy profiles of all studied antibiotic/sterol systems in lipid bilayer environments. Horizontal axes stands for distance between centers of masses between respective sterol and macrolactone ring. (**A**) Data for Candicidin D (CndD) and *iso*CndD, related to Amphotericin B (AmB). (**B**) Data for Partricin A (ParA) and *iso*ParA, related to AmB. (**C**) Data for Partricin B (ParB) and *iso*ParB, related to AmB.

Free energy profiles of formation of binary antibiotic/sterol complexes in respective membrane environments, resulting from Umbrella Sampling (US) approach, have indicated that in the case of all aromatic heptaene (AHM)/sterol combinations (CndD/Chol, CndD/Erg, ParA/Chol, ParA/Erg, ParB/Chol, ParB/Erg), a change in the mode of binding of a given *iso*AHM to respective sterol might be observed, in relation to the native AHM antibiotic. Shallow, local minima in the distance range of 5–6 angstroms, which could be observed in the free energy profiles of almost all native antibiotics, became the global minima in the free energy profiles of all *iso*AHMs (Figure 2A–C). This means that the centers of masses of *iso*AHM and sterol molecules approached closer to each other, which resulted in tighter binding. This mode of binding does not match a generally accepted model, where a sterol molecule approaches an antibiotic molecule by its heptaenic region. *Iso*AHMs preferably bind sterols at the cavity between conjugated double-bond system and the polyol chain, whereas the original AHMs should be facing a sterol molecule with the side opposite to its oxygenic functions (Figure S4).

In the case of CndD and ParB, isomerization of their *cis-trans* heptaenic chromophores to the *all-trans* versions did not have an observable impact on the binding energies between these antibiotics and both investigated sterols. On the contrary, for ParA, an interesting trend could be observed. In relation to the native ParA, *iso*ParA has slightly weakened its interactions with Chol, while deepening the global, energetic minimum of its binding to Erg. Nevertheless, those differences were not game-changers by any means, since deltas of ΔGs equal to ~2 kcal mol^−1^ were far from remarkable.

In the light of the aforementioned results, it was mandatory to conduct further numerical studies on single, aromatic heptaene molecules embedded in lipid bilayers in order to relate the spotted differences in their behavior—if any—to the shape of their chromophores and to the type of accompanying sterols. Considering the formation of transmembrane, potassium-evacuating channels to be the real mode of action of polyene macrolides—which seems to be proven quite strongly so far [22,23]—one could assume that a stable, vertical alignment of the major axis of an antibiotic molecule, inserted into membrane environment, should strongly contribute to the kinetics of channel formation. Therefore, major axes of six studied AHMs and of AmB were defined, using centers of masses of properly selected groups of atoms from the macrolactone rings. Later on, for all systems, evolution of variation of a given axis in relation the lipid bilayer normal (Z axis, perpendicular to a membrane system), was traced over time.

**Figure 3:**
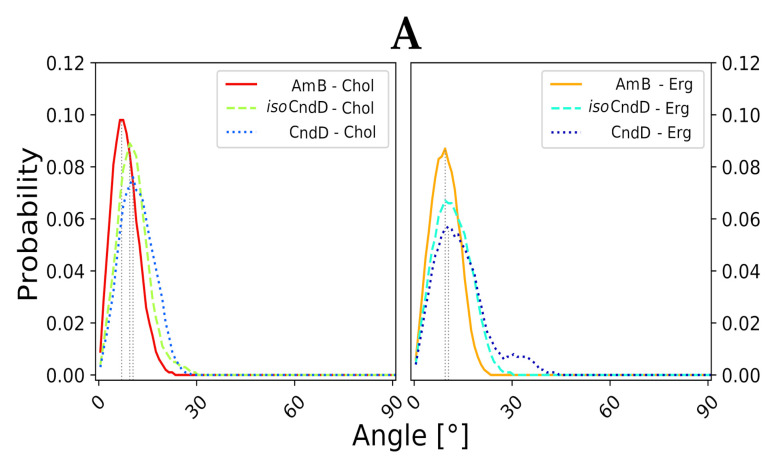

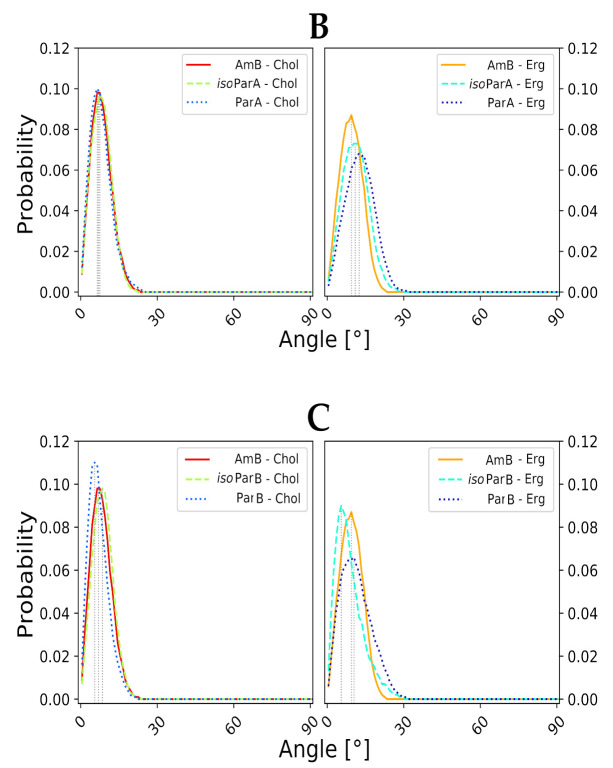
Distribution of angles, defined as variation of major axis of macrolactone ring of a given antibiotic in relation to the membrane normal (Z axis, perpendicular to lipid bilayer). Average variation angle for each system is represented by vertical dashed line. (**A**) Data for Candicidin D (CndD) and *iso*CndD, related to Amphotericin B (AmB). (**B**) Data for Partricin A (ParA) and *iso*ParA, related to AmB. (**C**) Data for Partricin B (ParB) and *iso*ParB, related to AmB.

Molecular dynamics experiments, conducted for all 12 studied AH/sterol systems (+2 AmB/sterol setups for reference) exposed a consistent pattern. The native versions of AHMs were slightly more vertical in Chol membranes than in Erg membranes. On the contrary, *iso*AHMs were a little more aligned with the Z axis in Erg environments than in Chol systems (Figure 3A–C). However, the differences in angle distributions were not large enough to call these observations anything more than a pattern. Yet, the results obtained for AmB were generally in line with the images containing data for iso-antibiotics, which could be related to the fact, that *iso*AHMs and AmB contain the same, *all-trans* heptaenic chromophores. Nevertheless, it was difficult not to notice that the angle probability (the high, structural resemblance of AmB’s and *iso*AHMs’ macrolactonic rings), one should relate the source of observed differences to the presence of an alkyl-aromatic side chain in the structure of AHMs, which AmB is deprived of. The distributions of the alignment of the alkyl-aromatic side chain of studied, single AHM molecules in lipid bilayers (upper leaf/lower leaf/in between) were quite chimeric and did not display any significant regularities. Moreover, no impact of the side chain on the mode of sterols’ binding was revealed so far.

## 3. Discussion

In this paper, experimental evidence for a markedly enhanced selective toxicity of *all-trans* isomers of three AHMs, obtained upon photochemical isomerization of Candicidin D, Partricin A (Gedamycin) and Partricin B (Vacidin), in comparison with the original *cis-trans* compounds, have been presented. There is little doubt that this quite spectacular improvement of selective toxicity was mainly due to the decreased hemolytic properties of the light-induced isomers of the aromatic heptaene antibiotics. The previously noted remarkably higher antifungal in vitro activity of original AHMs in BDS medium in comparison with that of Amphotericin B [15] was confirmed in our hands but not observed in RPMI 1640 medium under conditions recommended by CLSI [17]. The reason for such a difference is not known, although we can speculate that differences in pH values between both media (7.0 for buffered RPMI-1640 and 5.8 for non-buffered BDS) may matter.

Computational experiments, presented in this contribution, have proved that the differences in the mode of binding of aromatic heptaenes and isomeric aromatic heptaenes to cholesterol and ergosterol molecules in lipid bilayer environments were far from spectacular, although they exhibited some desirable patterns. In our opinion, these differences contribute in part to the observed enhanced selective toxicity of *all-trans* isomers comparing to their mother *cis-trans* antibiotic molecules but this input is moderate and must be supported by other factors. The slight contribution of parameters determined by Umbrella Sampling and molecular dynamics calculation to the overall biological effect does not indicate that molecular modelling studies were conducted erroneously. Rather, this points to the fact that attributing the enhancement of selective toxicity to the behavior of only a single antibiotic molecule and its binding to the molecular target was a little naive and that the case is more complex. One should consider whether the sterol molecules are the true molecular targets of AHMs. It seems, rather, that the lipid bilayers—containing Chol/Erg populations and treated as a whole—are in fact at the gunpoint of polyene macrolides. The presence of sterol molecules determines some crucial attributes of biological membranes, such as thickness and fluidity, and those attributes vary between Chol- and Erg-containing lipid bilayers. It seems reasonable to assume that a better match between a given antibiotic molecule and the membrane it targets should contribute to the antibiotic’s biological activity. For instance, the native *cis-trans* versions of AHMs, which contain shorter and more flexible macrolide rings, might ‘feel better’ in slightly thinner and more fluid cholesterol-containing membranes, whereas in the case of the isomeric, longer and rigid *all-trans* versions of AHs, a little tighter and less fluid ergosterol-containing membranes could comprise a more enjoyable environment. This pattern was suggested by data presented in Figure 3. Although, for single molecules, differences between deviations of the antibiotics’ major axes from the Z axes might seem negligible, it should be taken into account that channel-forming agents must—by definition—work as a team. Therefore, for several antibiotic molecules embedded in one bilayer, those small differences might sum up and result in more (or less) efficient formation of lethal pores.

The role of the alkyl-aromatic side chain of AHMs in their biological activity remains an open question. Umbrella Sampling experiments did not reveal its role in the mode of binding of sterol molecules. Nevertheless, one must consider that US is a one-dimensional experiment, hence the whole conformational space of AHM/sterol mutual orientations was not covered. To solve that issue, a set of two-dimensional metadynamics simulations are being currently conducted for AHMs’ and iso-AHMs’ complexes with Chol and Erg, in order to shed a new light on the alkyl-aromatic side chains’ role in interactions with the sterols.

Even if the side chains’ role in Chol/Erg binding were revealed, it would not be reasonable to assume that it is their only function. Aromatic heptaenes display up to two orders of magnitude higher antifungal activity in BDS medium than AmB and that brute force must be associated—at least partially—with the presence of the alkyl-aromatic side chains. Therefore, it seems crucial to consider other stages of AHMs’ molecular mode of action, where the side chain of a given macrolide antibiotic might strongly contribute to the agent’s high biological activity. Perhaps it serves as an anchor for the rest of the molecule, while entering a lipid bilayer? Or perhaps it plays a key role in assembling of two half-channels, embedded in opposite sides of a membrane, into a functional channel? Those questions are currently under our detailed investigations, incorporating spectroscopic and computational approaches.

## 4. Materials and Methods

### 4.1. Preparation and Isolation of Compounds

The crude Candicidin and Aureofacin (Partricin) complexes were supplied by the Pharmaceutical Work “Polfa”, Tarchomin, Poland.

#### 4.1.1. Isolation of ParA, ParB and CndD from Antibiotic Complexes

Isolation of ParA and ParB from the Aureofacin (Partricin) complex was performed by means of centrifugal partition chromatography (CPC) on a Gilson CPC-250 apparatus (Gilson Inc., Middleton, WI, USA), equipped with the Ecom flash 14 DAD 600 detector (Gilson Inc., Middleton, WI, USA) and Gilson fraction collector LS-5600 (Gilson Inc., Middleton, WI, USA). The biphasic liquid system: chloroform, methanol and borate buffer, pH = 8.2 (4:4:3 *v*/*v*/*v*) was prepared by mixing the respective portions of the solvents at room temperature. The separation was performed in ascending mode, using upper phase as the mobile phase at a flow rate of 5 mL min^−1^ and the rotational speed of 800 rpm. The effluent was monitored at a wavelength of 378 nm. A sample of the Partricin complex dissolved in the upper phase, 8 mg mL^−1^, was introduced via a manual injection valve and an injection loop of 10 mL.

Isolation of CndD from the Candicidin complex was performed by the sequential CPC and preparative HPLC. All the CPC chromatographic conditions were the same as described above. A fraction collected from the CPC experiment at R_T_ = 41 min was subjected to HPLC performed on a Teledyne ISCO apparatus ACCQPrep HP125 (Teledyne ISCO, Lincoln, NE, USA). The HPLC separation conditions were as follows: column LiChrospher 100 RP-18e (250 × 25 mm, 10 µm, Merck KGaA, Darmstadt, Germany); mobile phase composition: 38% acetonitrile/62% ammonium acetate buffer (5.5 mmol, pH = 4.5), *v*/*v*; flow rate 29.5 mL min^−1^; detection at 378 nm; room temperature. A sample collected from CPC was dissolved in 25% DMSO/75% methanol, *v/v* mixture at 10 mg mL^−1^, and injected in a volume of 2 mL.

#### 4.1.2. Photochemical *Cis-Trans* → *All-Trans* Isomerisation of Components of Aureofacin and Candicidin Complexes and Isolation of *All-Trans* Isomers of ParA, ParB and CndD

The Candicidin and Aureofacin antibiotic complexes were dissolved at 5 mg mL^−1^ in a 95% methanol/5% water mixture and subjected to irradiation by monochromatic light of wavelength λ = 365 nm at room temperature for 2.5–3 h. This wavelength was chosen on the basis of the previous work [16] and was not optimized for this study. The progress of the photochemical isomerization reaction was monitored using UV–Vis spectroscopy and RP-HPLC analysis. The RP-HPLC analysis conditions were as follows: column: Luna 100 C18 (2) (150 × 4.6 mm, 5 µm); mobile phase composition: 38% acetonitrile/62% ammonium acetate buffer (5.5 mmol, pH = 4.5), *v/v*; flow rate 1 mL min^−1^; detection at 378 nm; room temperature.

Isolation of *all-trans* isomers from the post-reaction mixtures was performed by means of CPC and preparative HPLC, according to the procedure described above for isolation of CndD from Candicidin complex.

#### 4.1.3. Structural Analysis

##### NMR Experimental

The NMR spectra were recorded with a Bruker Ascend 700 MHz spectrometer equipped with a QCI CryoProbe. All the experiments were performed in the solvent system pyridine-*d_5_*/methanol-*d_4_* (9:1, *v*/*v*) with sample concentration of 10 mg mL^−1^. The one-dimensional ^1^H spectra were collected with digital resolution of 0.5 Hz. Two-dimensional ^1^H spectra were measured in the phase-sensitive mode with a spectral width of 7350 Hz. The DQF-COSY spectra were acquired in a 6080 × 512 matrix with 32 accumulations per increment and were processed in a 4K × 2K matrix.

##### HPLC-MS Analysis

The LC-DAD-MS system consisted of a liquid chromatograph, a degasser, a binarypomp, an auto-sampler, and a column oven was combined with a diode array detector (DAD) and MS detector with an electrospray source (AJS ESI) and quadrupole analyzer(1260 Infinity II and 6470 Triple Quad LC/MS, Agilent Technologies, Waldbronn, Germany). The Agilent ChemStation software (Agilent Technologies, Waldbronn, Germany) was used to control the LC-MS system and for data processing. The column effluent passed a DAD before arriving in the MS interface. Conditions of chromatographic separation: Luna 100 C18(2) column (150 × 4.6 mm, 5 µm); mobile phase composition: 38% acetonitrile/62% ammonium acetate buffer (5.5 mmol, pH = 4.5), *v*/*v*; flow rate 1 mL min^−1^; detection at 378 nm; room temperature.

The electrospray source was operated in a positive mode and the interface conditionwere as follows: Gas temperature 300 °C and flow 5 L/min, sheath gas temperature 250 °C and flow 11 L/min, nebulizer 45 psi, capillary voltage of 3500 V. The data were collected in a MS scanning mode (MS2 SCAN) with the range 150–1500 (*m/z*).

### 4.2. Microbial Strains and Culture Conditions

The reference strains used in this study were: *Candida albicans* ATCC 10231, *Candida glabrata* DSM 11226, *Candida krusei* DSM 6128, *Candida parapsilosis* DSM 5784, *Candida dubliniensis* CBS 7987, *Candida guilliermondii* DSM 11947, *Candida famata* DSM 3428, *Candida rugosa* DSM 2031, *Saccharomyces cerevisiae* ATCC 9763. Strains were grown at 30 °C in YPD medium (2% glucose, 1% Yeast Extract and 1% Bacto Peptone) and stored on YPD + 2% agar plates.

### 4.3. Antifungal In Vitro Activity Determination

Susceptibility testing was performed in two growth media: a) RPMI-1640 w/o sodium bicarbonate, with L-glutamine (Sigma-Aldrich, St. Louis, MO, USA) + 2% glucose + 3.45% MOPS/3-(*N*-morpholino)propanesulfonic acid/, pH adjusted to 7.0; b) BDS medium (1% Bacto Peptone + 2% glucose + 0.5% NaCl). The in vitro growth inhibitory activity of antifungals was quantified by determination of MIC values by the serial two-fold dilution method, using the 96-well microtiter plates. Serial dilutions of antifungals were prepared in the 16–0.03 µg mL^−1^ (RPMI-1640 medium) or 1–0.0015 µg mL^−1^ (BDS medium) range.

Conditions of the RPMI-1640-based assay were the same as outlined in the CLSI rec-ommendations [17], except for the end-point readout that was conducted by spectrophotometric determination of cell density at 660 nm, instead of the CLSI-recommended visual reading. Turbidity in individual wells was measured with a microplate reader (Victor^3^; Perkin Elmer, Waltham, MA, USA).

The 96-well microtiter plates were also used for determination of in vitro growth inhibitory activity in BDS medium. Individual wells were inoculated with 5 × 10^3^ cells mL^−1^ from the overnight culture in YPD medium. The plates were incubated at 37 °C for 24 h and then turbidity was measured with a microplate reader at 660 nm, as described above for the RPMI-1640-based assay.

MIC was defined as the lowest drug concentration in the particular well, for which the A_660_ value was not higher than 10% of that measured for the drug-free control.

### 4.4. Hemolytic Activity Determination

Red blood cell concentrates were kindly provided by the Regional Center for Blood Donation and Blood Treatment in Gdańsk. The hemolytic activity determination was carried out by the serial dilutions method, according to the procedure described earlier [24]. Human erythrocytes were suspended in the solution of saline to obtain a cell density of suspension 2 × l0^7^ cells per mL. Suitable amounts of diluted in DMSO solutions of compounds were added to the cell suspension in tubes and were incubated at 37 °C for 30 min and then centrifuged (1700× *g*, 5 min, 4 °C). The concentration of hemoglobin in supernatant after centrifugation of erythrocytes suspension was determined by measuring the absorbance at wavelength λ = 540 nm (A_540_^sample^ ). Control experiments with the same amount of DMSO were also performed (A_540_ ^DMSO^). The maximum level of hemolysis was obtained after incubation of cells suspension in the presence of 0.1% Triton X-100 (positive control, A_540_ ^0.1%Triton X-100^). Normal saline (0.9%) was used as negative control (0% lysis, A_540_ ^0.9%NaCl^). The EH_50_ values for each compound was calculated with the GraphPad Prism software (GraphPad Software Inc., San Diego, CA, USA). The EH_50_ is the interpolated concentration of compound, for which the A_540_ value is exactly 50% of the A_540_ value measured for the positive control sample. EH (%) was calculated as follows:

EH (%) = ((A_540_ ^sample^−A_540_ ^DMSO^)/(A_540_ ^0.1%Triton X−100^−A_540_ ^0.9%NaCl^)) × 100

### 4.5. Molecular Modelling

The behavior of three, native (*cis-trans*) representatives of AHs: candicidin D (CndD), partricin A (ParA) and partricin B (ParB) molecules and their isomerized (*all-trans*) versions in lipid bilayers was examined, as well as the free energy profiles of interactions between membrane-embedded AH molecules and selected sterols were studied. For a given native and isomerized antibiotic, lipid bilayers in two varieties: (i) a dipalmitoylphosphatidylcholine (DDPC) membrane with ~30 mol% of cholesterol (Cho) and (ii) a DPPC membrane with ~30 mol% of ergosterol (Erg) were used.

#### 4.5.1. Parametrization

The basic forcefield parameters for all AHs’ atomistic models were taken from CHARMM36 Generalized ForceField [25] using the web interface available at paramchem.org, accessed on 14 September 2020. Hence, these parameters were refined using GAUSSIAN09 software (Gaussian Inc., version 09, revision D.01, Wallingford, CT, USA) [26]. Firstly, the partial atomic charges for both molecules were recalculated ab initio on the MP2/6-31G* level of theory. Secondly, several dihedral angles which scored high penalties during the application of CHARMM36 forcefield, namely: those defined by the heavy atoms of the C35-C4 fragment of both molecules, were re-parameterized. This was performed using GAUSSIAN09 implementation of a dihedral scanner on a MP2/6-31G* level of theory with a dihedral step of 5 degrees. The resulting energetic profiles were translated to the CHARMM36 language using ForceField ToolKit (ffTK) [27] as a part of VMD [28]. Additionally, parameters defining the properties of the two dihedrals constituting the glycosidic bond between the aglycone and the mycosamine moiety were taken from our previous studies on amphotericin B, as these fragments of CndD, *iso*CndD, ParA, *iso*ParA, ParB and *iso*ParB were structurally identical to the one of AmB.

#### 4.5.2. System Preparation

All 14 simulated systems: candicidin D with cholesterol, candicidin D with ergosterol, *iso*candicidin D with cholesterol, *iso*candicidin D with ergosterol, partricin A with cholesterol, partricin A with ergosterol, *iso*partricin A with cholesterol, *iso*partricin A with ergosterol, partricin B with cholesterol, partricin B with ergosterol, *iso*partricin B with cholesterol, *iso*partricin B with ergosterol, amphotericin B with cholesterol and amphotericin B with ergosterol were constructed with the CHARMM Membrane Builder [29]. A single molecule of every studied polyene macrolide antifungal antibiotic was embedded in a lipid bilayer composed of 48 cholesterol or 42 ergosterol molecules and 112/98 DPPC molecules, respectively, solvated with approximately ~4400 water molecules and 14 K^+^ and Cl^−^ ions, corresponding to concentration of 0.15 mol/dm^3^ to provide physiological ionic strength. The CHARMM36 forcefield was used for the antibiotics, sterols and DPPC molecules, while the TIP3P model [30] was used for water.

#### 4.5.3. Molecular Dynamics Simulations

All simulations were implemented in GROMACS 2020.4 (The GROMACS development teams at the Royal Institute of Technology and Uppsala University, version 2020.4, Stockholm/Uppsala, Sweden) [31] containing plumed 2.6.2 plugin [32]. The simulations were performed in NPT ensemble with the temperature kept at 310 K by Nose-Hoover dynamics and pressure kept at 1 Bar with the Parrinello-Rahman method [33]. Particle Mesh Ewald algorithm was used for a long range electrostatics interactions with real-space cutoff of 10 Å [34]. Van der Waals interactions were evaluated using a (Force-switch/smooth) cutoff of 12 Å with a switching radius of 10 Å. The equations of motion were integrated with the velocity Verlet algorithm with a time step of 2 fs. After the systems were equilibrated in seven steps according to the procedure described in the CHARMM Membrane Builder, a 500 ns production runs, with a 2 fs time step in conjunction with the LINCS algorithm, were performed for each system.

#### 4.5.4. Umbrella Sampling

The Umbrella Sampling (US) method [35] was used to obtain the free energy profiles of antibiotic/sterol interactions in lipid bilayers. Working reaction coordinate was defined as the distance between the center of masses of a given macrolide ring and a sterol ring system, projected on the xy-plane. The initial structures for the US calculations were selected from a 100 ns long steered-MD simulations, in which the distance was moved from 5 to 15 Å with harmonic restraint build up with the force constants equal to 1.2 kcal/(mol × Å2). The reaction coordinate was divided into 10 equally sized windows (5 ≤ z ≤ 15 Å) with 1Å spacing and restrained with harmonic potential with the force constant equal to 0.6 kcal/(mol∙Å). Each window was first equilibrated for 100 ns and then simulated for 1000 ns. The sampling in each window was improved by replica exchange method with exchange between successive windows. The free energy profiles were calculated using the standard weighted histogram analysis method (WHAM) [36].

## 5. Conclusions

The *all-trans* isomers of three representative AHMs demonstrate lower toxicity against human erythrocytes than the mother antibiotics and in consequence, a substantially improved in vitro selective toxicity, manifested by the higher EH_50_/MIC values. Molecular modeling simulations have revealed slight differences between AHMs and *iso*-AHMs in geometries of heptaene:sterol complexes and polyene macrolide molecule alignment in cholesterol- and ergosterol-containing membranes, that contribute in part to this effect. In order to see the full picture, it seems necessary to take into account the interactions of multiple AHM molecules with sterol-containing membranes as their targets.

## Figures and Tables

**Table 1:** Antifungal in vitro activity of Candicidin D (CndD), Partricin A (ParA), Partricin B (ParB), their *all-trans* isomers, *iso*CndD, *iso*ParA, *iso*ParB and Amphotericin B (AmB). MIC (minimal inhibitory concentration) values were determined in RPMI-1640 medium by the serial two-fold dilution method, using the 96-well microtiter plates.

Compound	CndD	*iso*CndD	ParA	*iso*ParA	ParB	*iso*ParB	AmB
Microorganism	MIC (µg mL^−^^1^)						
*C. albicans*	0.25	0.5	0.25	1.0	0.25	0.5	0.25
*C. glabrata*	0.5	0.5	0.125	0.5	0.125	0.25	1.0
*C. krusei*	0.25	0.25	0.125	0.5	0.5	0.5	0.5
*C. parapsilosis*	0.25	0.06	0.06	0.125	0.25	0.125	0.5
*C. dubliniensis*	0.25	0.125	0.125	0.5	0.25	0.25	0.25
*C. guilliermondii*	1.0	0.5	0.5	0.5	1.0	1.0	0.25
*C. famata*	1.0	1.0	1.0	1.0	1.0	1.0	1.0
*C. rugosa*	1.0	1.0	1.0	1.0	1.0	1.0	2.0
*S. cerevisiae*	0.5	0.25	0.125	0.5	0.125	0.25	0.5

**Table 2:** Antifungal in vitro activity of Candicidin D (CndD), Partricin A (ParA), Partricin B (ParB), their *all-trans* isomers *iso*CndD, *iso*ParA, *iso*ParB and Amphotericin B (AmB). MIC (minimal inhibitory concentration) values were determined in BDS medium by the serial two-fold dilution method, using the 96-well microtiter plates.

Compound	CndD	*iso*CndD	ParA	*iso*ParA	ParB	*iso*ParB	AmB
Microorganism	MIC (µg mL^−^^1^)						
*C. albicans*	0.003	0.003	0.002	0.006	0.002	0.003	0.06
*C. glabrata*	0.006	0.003	0.002	0.006	0.002	0.006	0.25
*C. krusei*	0.006	0.003	0.002	0.006	0.003	0.003	0.25
*C. parapsilosis*	0.01	0.006	0.003	0.006	0.006	0.006	0.125
*C. dubliniensis*	0.002	0.002	0.001	0.006	0.002	0.002	0.06
*C. guilliermondii*	0.03	0.03	0.006	0.03	0.01	0.01	0.125
*C. famata*	0.01	0.03	0.01	0.05	0.01	0.05	0.25
*C. rugosa*	0.03	0.03	0.03	0.05	0.03	0.03	0.125
*S. cerevisiae*	0.006	0.006	0.002	0.001	0.003	0.003	0.06

**Table 3:** Hemolytic activity of Candicidin D (CndD), Partricin A (ParA), Partricin B (ParB), their *all-trans* isomers *iso*CndD, *iso*ParA, *iso*ParB and Amphotericin B (AmB). EH_90_ (erythrocyte hemolysis) values were determined under conditions described in 4.4. Each value is a mean of three independent determinations ± SD.

Compound	CndD	*iso*CndD	ParA	*iso*ParA	ParB	*iso*ParB	AmB
EH_50_ (µg mL^−1^)	7.53 ± 0.82	16.67 ± 1.40	1.28 ± 0.68	>20	0.66 ± 0.09	9.64 ± 1.79	3.46 ± 0.15

**Table 4:** Selective toxicity indexes (STIs) of Candicidin D (CndD), Partricin A (ParA), Partricin B (ParB), their *all-trans* isomers *iso*CndD, *iso*ParA, *iso*ParB and Amphotericin B (AmB). STI = EH_50_/MIC against *C. albicans* in RPMI-1640 medium.

Compound	CndD	*iso*CndD	ParA	*iso*ParA	ParB	*iso*ParB	AmB
STI	30.12	33.34	5.12	>20	2.64	19.28	13.84

## Supplementary Materials

The following are available online at https://www.mdpi.com/article/10.3390/ijms221810108/s1, Figure S1A–F: HPLC chromatograms of preparations of CndD, ParA, ParB and their *all-trans* isomers.; Figure S2A–F: 1H NMR spectra of CndD, ParA, ParB and their *all-trans* isomers.; Figure S3A–F: Fragments of 2D DOF-COSY 1H NMR spectra of CndD, ParA, ParB and their *all-trans* isomers.; Figure S4: Structures of the AH/sterol complexes.
