# Supplementary material for: Quest for the Molecular Basis of Improved Selective Toxicity of All-Trans Isomers of Aromatic Heptaene Macrolide Antifungal Antibiotics
Source: Int J Mol Sci. 2021 Sep 18;22(18):10108. doi: 10.3390/ijms221810108 (PMC8468583; doi:10.3390/ijms221810108)
Supplement: Supplementary file 1 [file ijms-22-10108-s001.zip › ijms-1377958-SI.pdf]

Supplementary materials

Figures S1A-F. HPLC-DAD-ESIMS chromatograms of CndD, ParA, ParB, isoCndD, isoParA and isoParB

Figure S1A

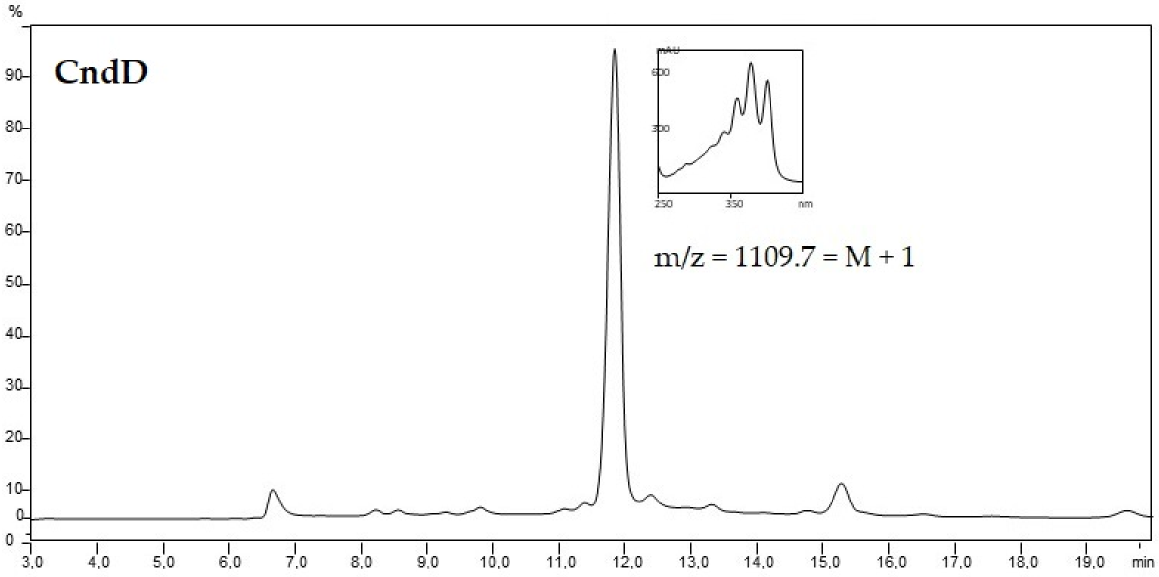

HPLC-DAD-ESIMS chromatogram of Candicidin D sample.
Chromatographic conditions: column Luna 100 C18(2), (150 mm × 4.6 mm, 5 µm). Mobile phase composition: 38% acetonitrile/ 62% ammonium acetate buffer (5.5 mmol, pH = 4.5), v/v; at a flow rate of 1 mL/min; inj. volume = 10 µL. Detection at 378 nm, room temperature.

Figure S1B

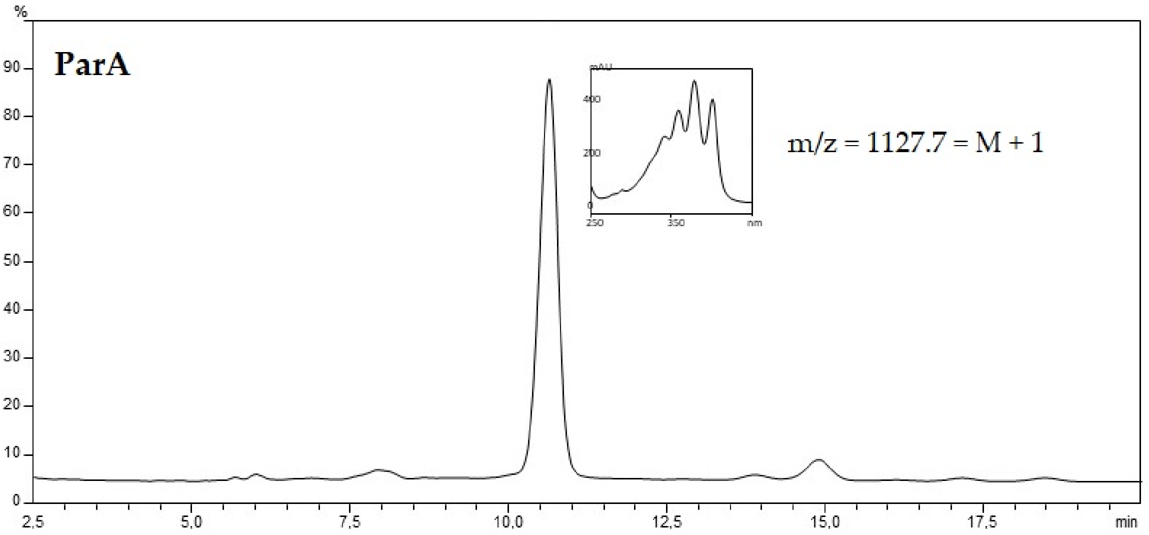

HPLC-DAD-ESIMS chromatogram of Partricin A sample.
Chromatographic conditions: column Luna 100 C18(2), (150 mm × 4.6 mm, 5 µm). Mobile phase composition: 38% acetonitrile/ 62% ammonium acetate buffer (5.5 mmol, pH = 4.5), v/v; at a flow rate of 1 mL/min; inj. volume = 10 µL. Detection at 378 nm, room temperature.

Figure S1C

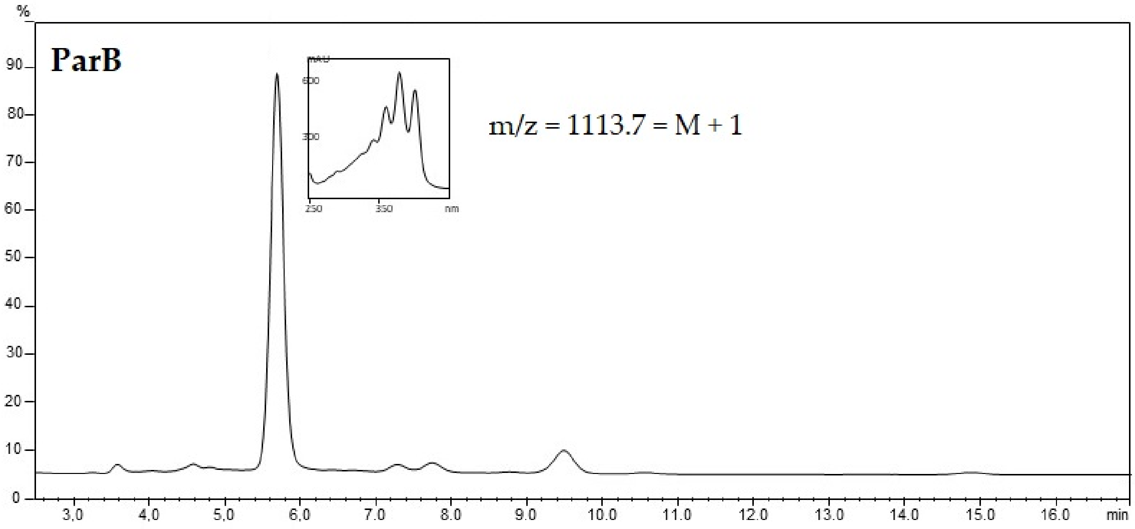

HPLC-DAD-ESIMS chromatogram of Partricin B sample.
Chromatographic conditions: column Luna 100 C18(2), (150 mm × 4.6 mm, 5 µm). Mobile phase composition: 38% acetonitrile/ 62% ammonium acetate buffer (5.5 mmol, pH = 4.5), v/v; at a flow rate of 1 mL/min; inj. volume = 10 µL. Detection at 378 nm, room temperature.

Figure S1D

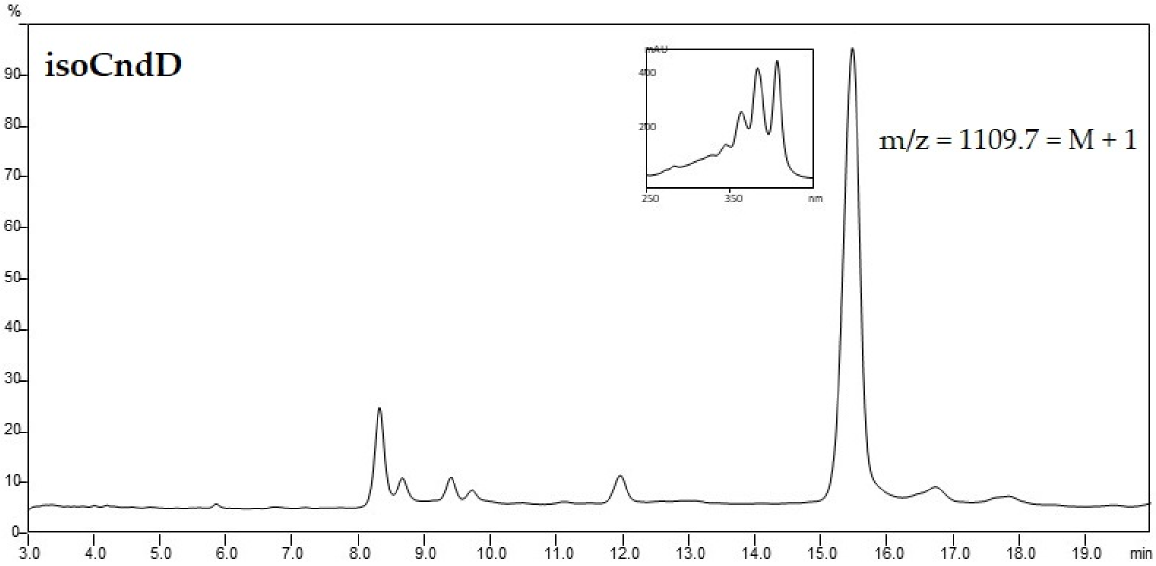

HPLC-DAD-ESIMS chromatogram of isoCandicidin D sample.
Chromatographic conditions: column Luna 100 C18(2), (150 mm × 4.6 mm, 5 µm). Mobile phase composition: 38% acetonitrile/ 62% ammonium acetate buffer (5.5 mmol, pH = 4.5), v/v; at a flow rate of 1 mL/min; inj. volume = 10 µL. Detection at 378 nm, room temperature.

Figure S1E

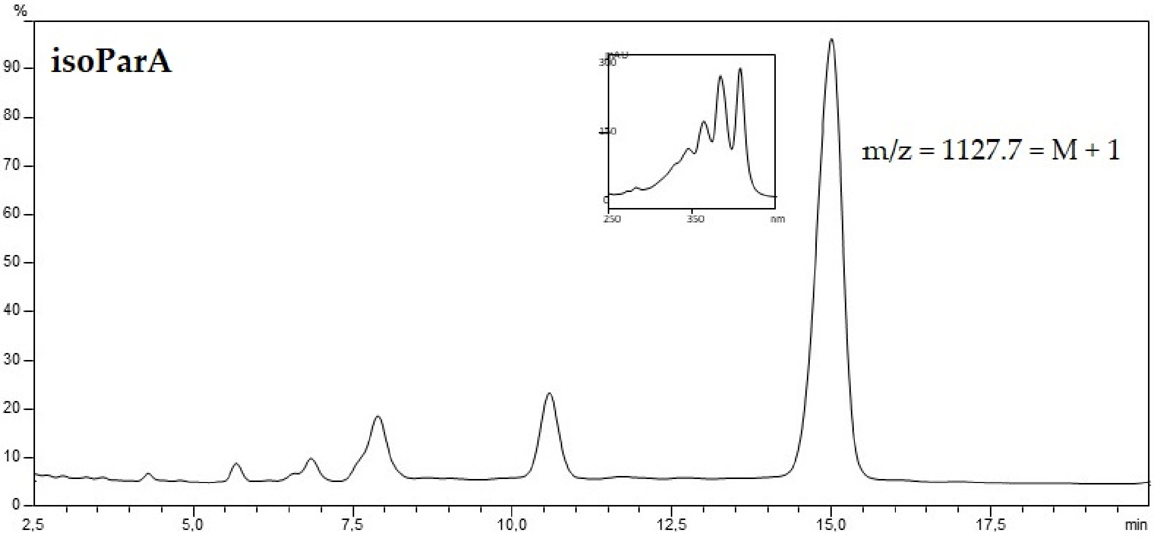

HPLC-DAD-ESIMS chromatogram of isoPartricin A sample.

Chromatographic conditions: column Luna 100 C18(2), (150 mm × 4.6 mm, 5 µm). Mobile phase composition: 38% acetonitrile/ 62% ammonium acetate buffer (5.5 mmol, pH = 4.5), v/v; at a flow rate of 1 mL/min; inj. volume = 10 µL. Detection at 378 nm, room temperature.

Figure S1F

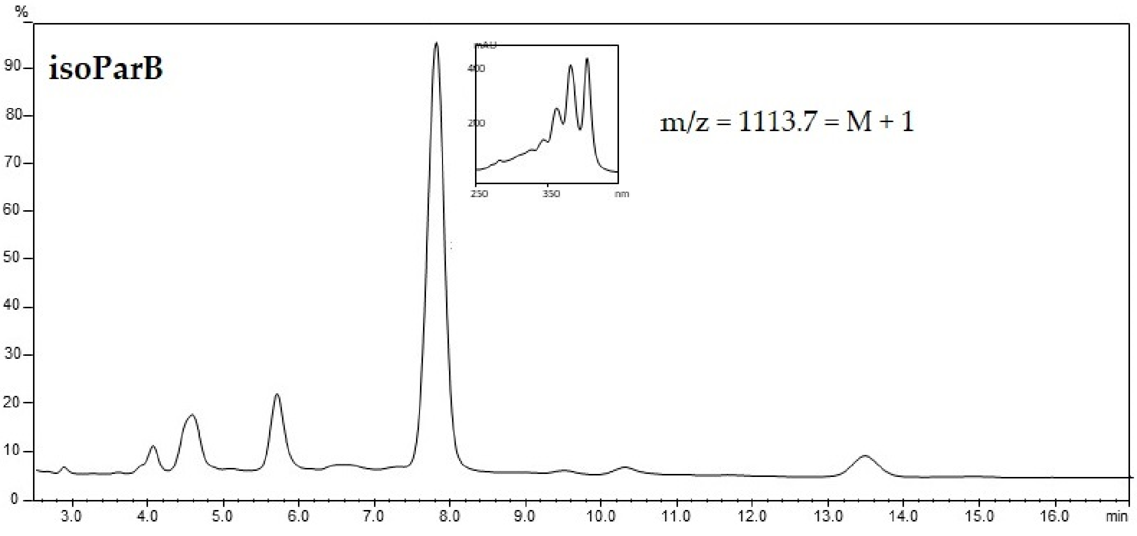

HPLC-DAD-ESIMS chromatogram of isoPartricin B sample

Chromatographic conditions: column Luna 100 C18(2), (150 mm × 4.6 mm, 5 µm). Mobile phase composition: 38% acetonitrile/ 62% ammonium acetate buffer (5.5 mmol, pH = 4.5), v/v; at a flow rate of 1 mL/min; inj. volume = 10 µL. Detection at 378 nm, room temperature.

Figures S2A-F. 1H NMR spectra of CndD, ParA, ParB, isoCndD, isoParA and isoParB

Figure S1A

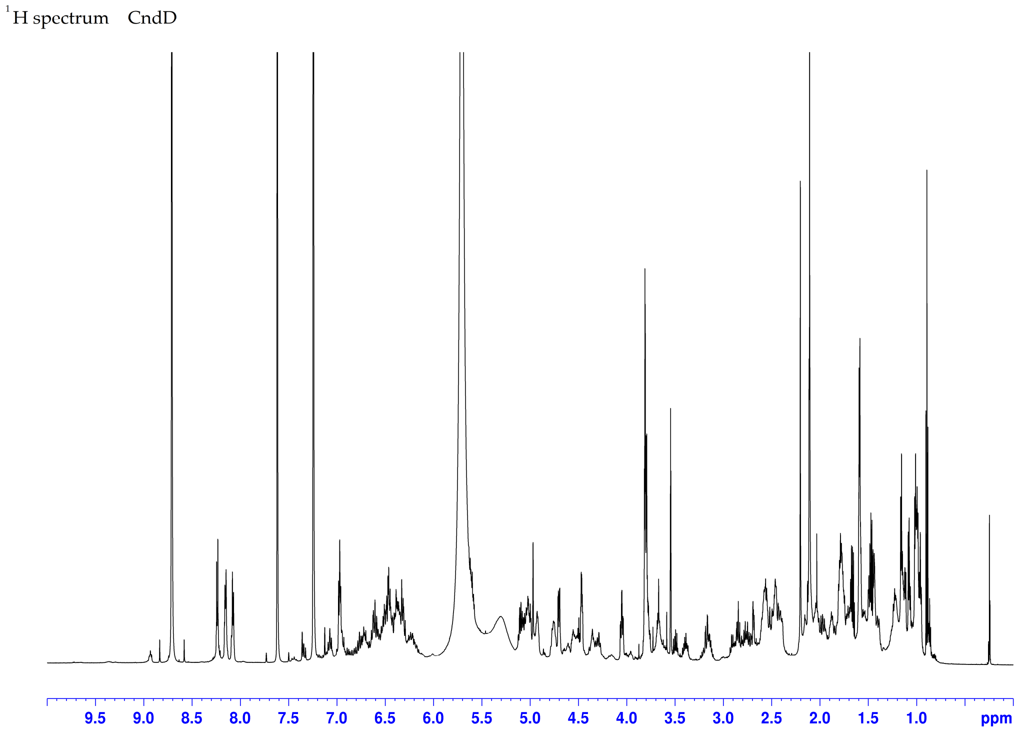

Figure S2B

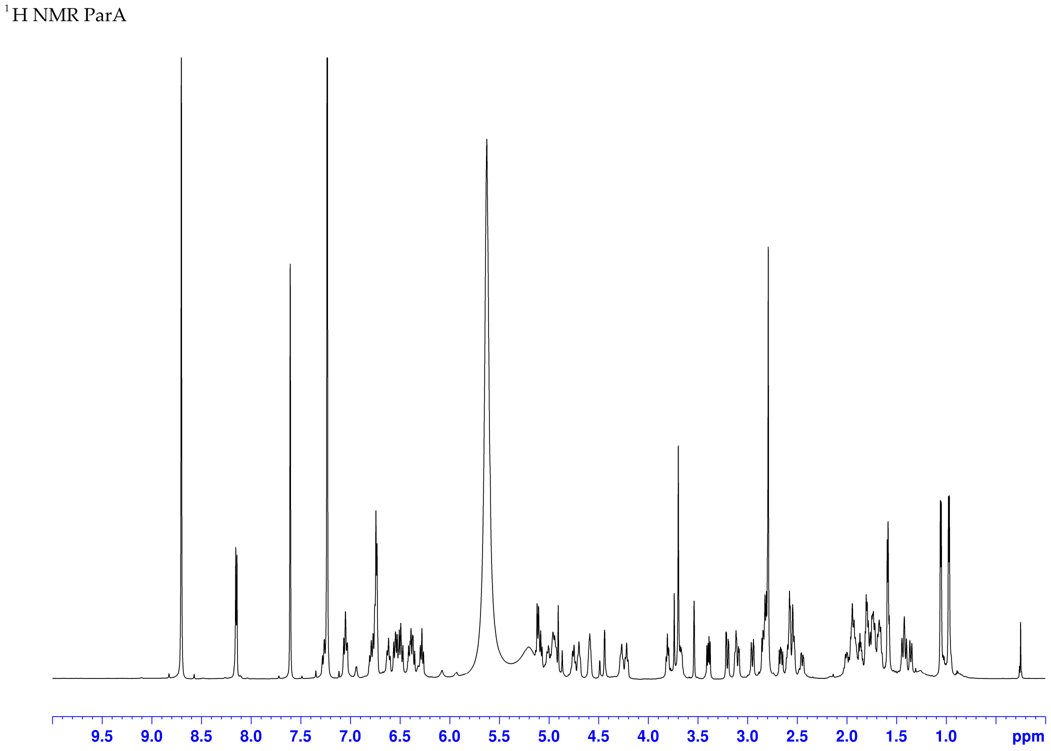

Figure S2C

^{1}H NMR ParB

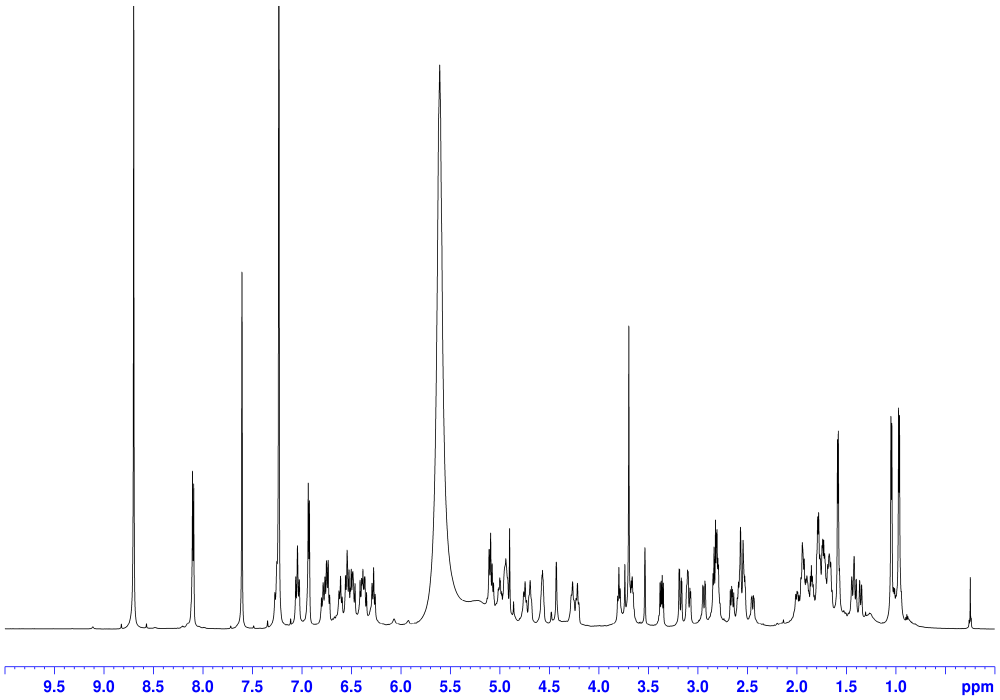

Figure S2D

^{1}H NMR isoCndD

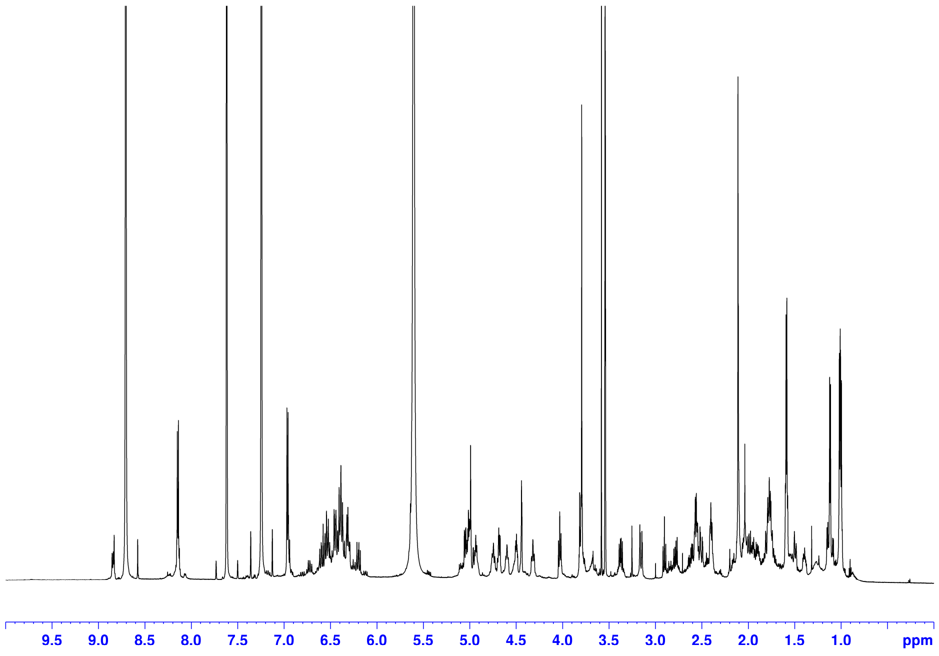

Figure S2E

^{1}H NMR isoParA

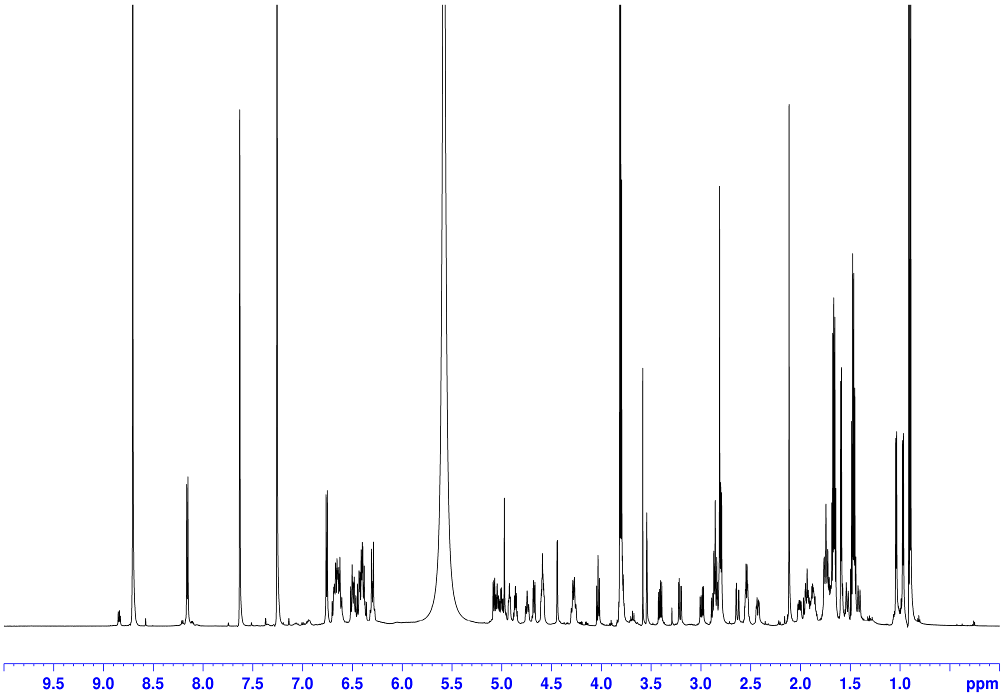

Figure S2F

^{1}H NMR isoParB

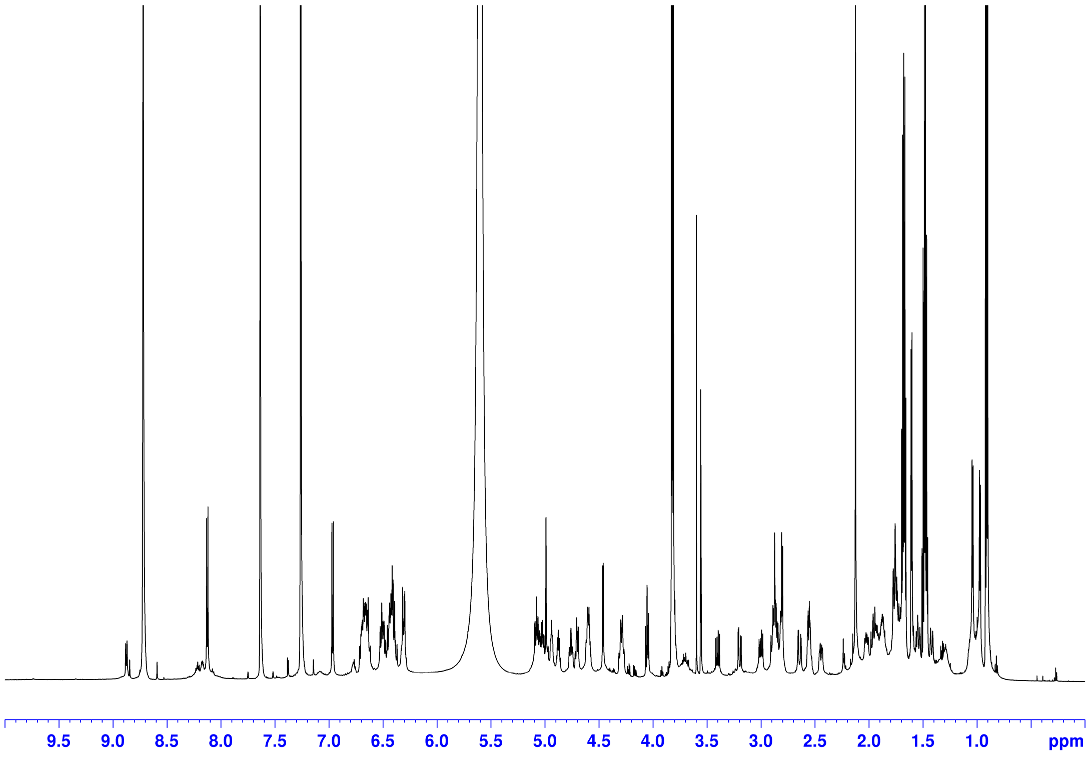

Figures S3A-F. Fragments of 2D DOF-COSY 1H NMR spectra of CndD, ParA, ParB and their *all-trans* isomers

Figure S3A

2D ^{1}H NMR DOF-COSY spectrum fragment CndD

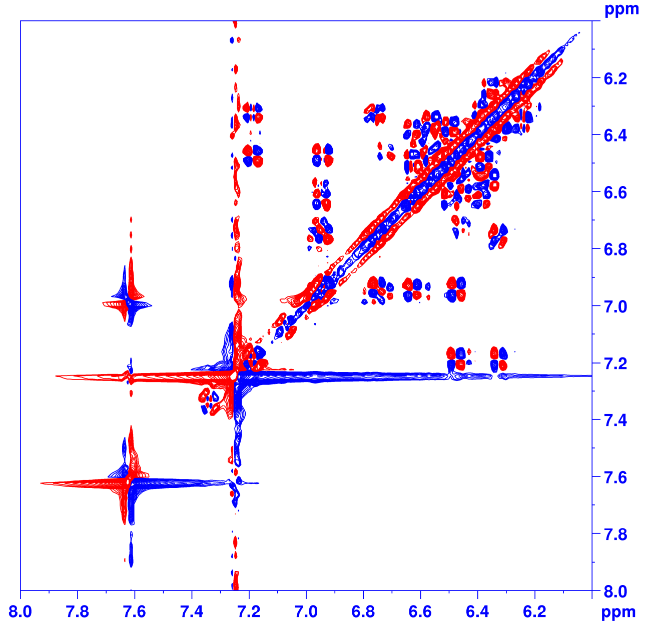

Figure S3B

2D ^{1}H NMR DOF-COSY spectrum fragment isoCndD

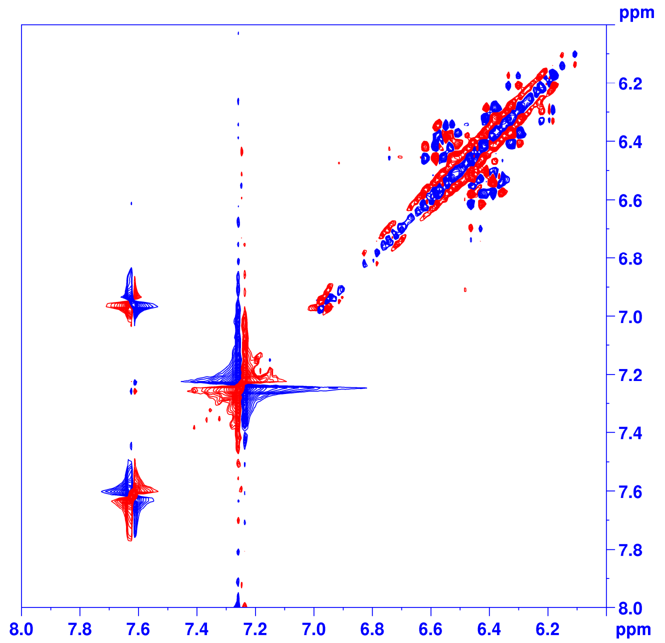

Figure S3C

2D ^{1}H NMR DOF-COSY spectrum fragment ParA

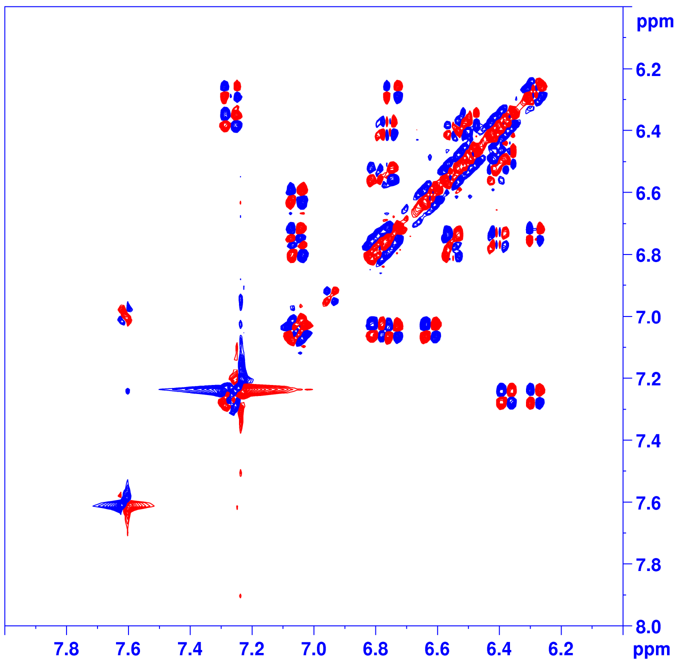

Figure S3D

2D ^{1}H NMR DOF-COSY spectrum fragment iso ParA

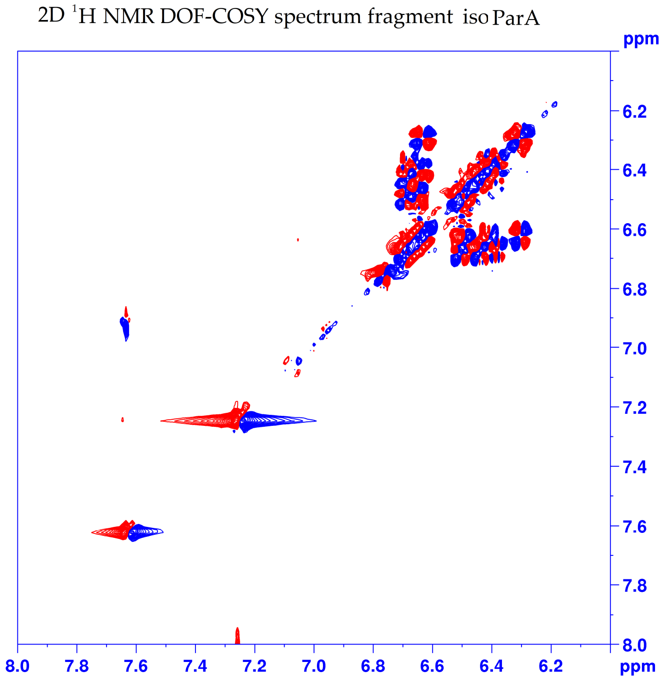

Figure S3E

2D ^{1}H NMR DOF-COSY spectrum fragment ParB

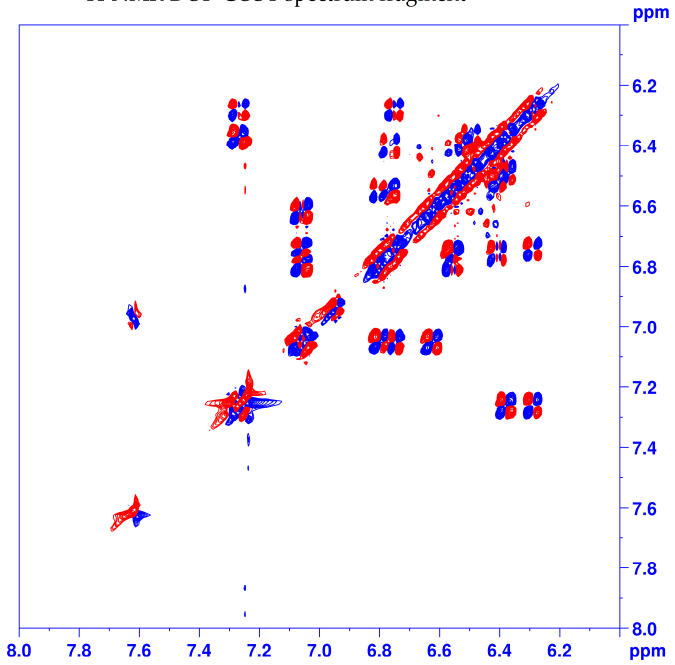

Figure S3F

2D ^{1}H NMR DOF-COSY spectrum fragment isoParB

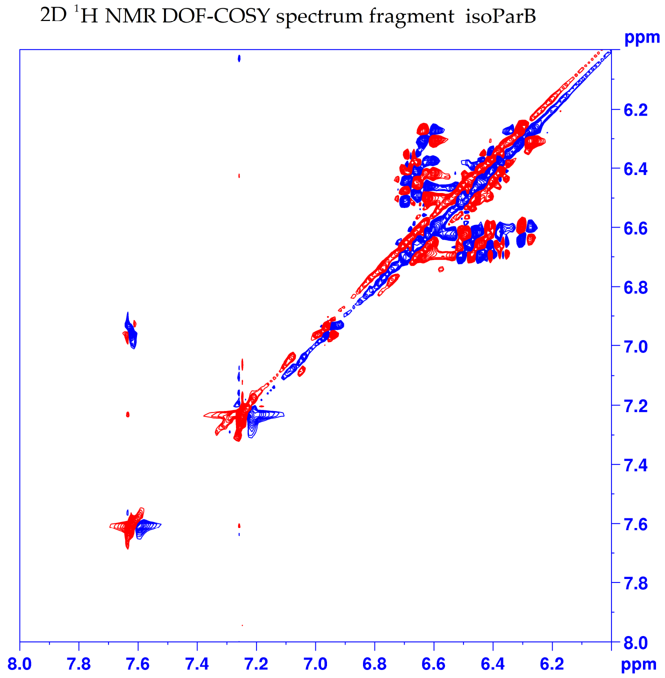

Figure S4. Structures of the AHM/sterol complexes, corresponding to the closer (1: 5-6 Å) and further (2: 7-8 Å) minima of the free energy profiles, obtained by cluster analysis of the resulting US trajectories.

	Chol		Erg	
	1	2	1	2
isoCndD	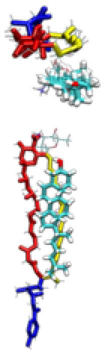	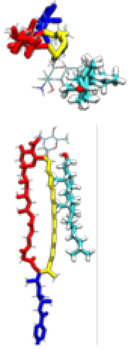	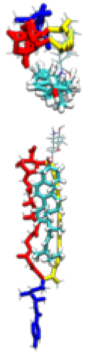	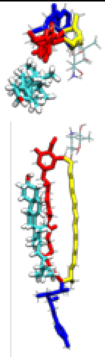 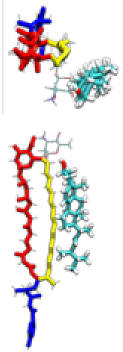
CndD	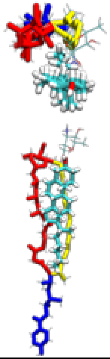	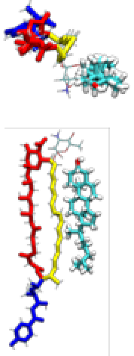	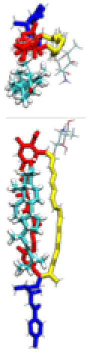	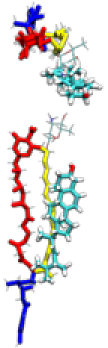

	Chol		Erg	
	1	2	1	2
isoParA	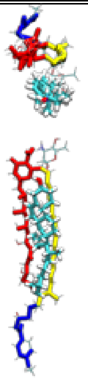	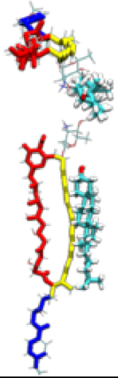 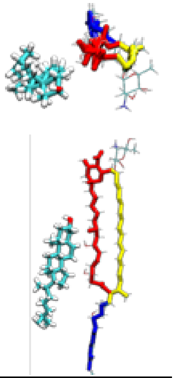	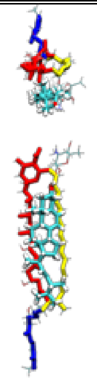	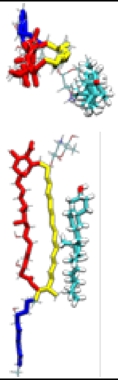
ParA	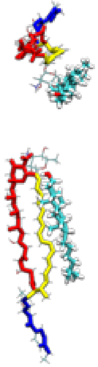		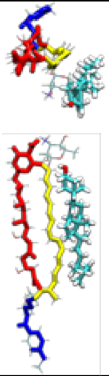	

	Chol			Erg		
	1	2		1	2	
isoParB	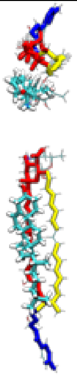			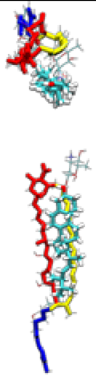	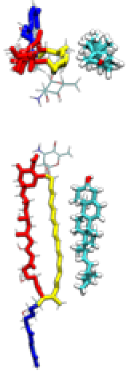	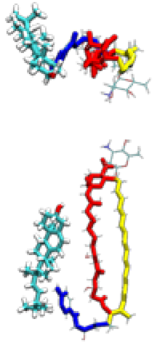
ParB	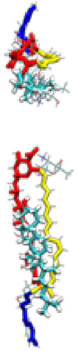	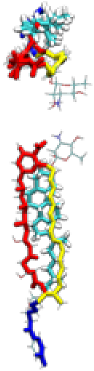	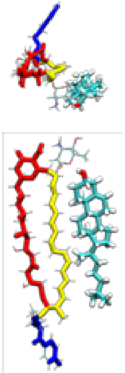	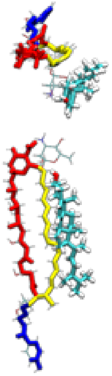		
